# Daily Temperature Fluctuations Alter Interactions between Closely Related Species of Marine Nematodes

**DOI:** 10.1371/journal.pone.0131625

**Published:** 2015-07-06

**Authors:** Nele De Meester, Giovanni A. P. Dos Santos, Annelien Rigaux, Yirina Valdes, Sofie Derycke, Tom Moens

**Affiliations:** 1 Marine Biology Unit, Department of Biology, Ghent University, Gent, Belgium; 2 Center for Molecular Phylogeny and Evolution, Ghent University, Gent, Belgium; 3 Departmento de Zoologia, CCB, Federal University of Pernambuco, UFPE, Recife-PE, Brasil; University of Connecticut, UNITED STATES

## Abstract

In addition to an increase in mean temperature, climate change models predict decreasing amplitudes of daily temperature fluctuations. In temperate regions, where daily and seasonal fluctuations are prominent, such decreases in daily temperature fluctuations can have a pronounced effect on the fitness of species and on the outcome of species interactions. In this study, the effect of a temperature regime with daily fluctuations versus a constant temperature on the fitness and interspecific interactions of three cryptic species of the marine nematode species complex of *Litoditis marina* (Pm I, Pm III and Pm IV) were investigated. In a lab experiment, different combinations of species (monospecific treatment: Pm I and Pm IV and Pm III alone; two-species treatment: Pm I + Pm IV; three-species treatment: Pm I + Pm IV + Pm III) were subjected to two different temperature regimes: one constant and one fluctuating temperature. Our results showed that fluctuating temperature had minor or no effects on the population fitness of the three species in monocultures. In contrast, interspecific interactions clearly influenced the fitness of all three species, both positively and negatively. Temperature regime did have a substantial effect on the interactions between the species. In the two-species treatment, temperature regime altered the interaction from a sort of mutualism to commensalism. In addition, the strength of the interspecific interactions changed depending on the temperature regime in the three-species treatment. This experiment confirms that interactions between the species can change depending on the abiotic environment; these results show that it is important to incorporate the effect of fluctuations on interspecific interactions to predict the effect of climate change on biodiversity.

## Introduction

Temperature is one of the most important environmental factors affecting many aspects of the life cycles of species (e.g. development and growth rates, body size, reproduction, etc.), and is considered an important selective agent [1]. Over the past 100 years, global temperature has increased by approximately 0.6°C.

Climate change models not only predict rising average temperatures, but also an increasing frequency of episodic temperature extremes [2] and decreasing amplitudes in daily temperature fluctuations [3]. In temperate regions, where daily and seasonal fluctuations are prominent, such decreases in daily temperature fluctuations can have a pronounced effect on the fitness of species as well as on the outcome of species interactions. For instance, lower maximum peak temperatures can have species-specific effects on development rate, survival and reproduction of individual species. Such effects are difficult to predict, since both increased and decreased development rates have been observed under a fluctuating compared to a constant temperature regime [4,5]. In addition, a higher mortality and lower reproduction rate at constant temperature have frequently been found [6,7]. Species-specific responses to daily fluctuations can potentially influence species interactions in three ways: directly, by changing the competitive abilities of species; indirectly, as a result of changes in population dynamics of one of the species which indirectly influences other species (e.g. by food depletion); or by a combination of both direct and indirect processes [8]. Moreover, if species respond differentially to environmental fluctuations, daily temperature cycles can contribute to a stable coexistence between species [9]. Closely related species are expected to have high competition [10], and changes in temperature fluctuations may therefore lead to changes in interspecific interactions and facilitate the co-occurrence of species.

An intriguing case of coexistence is that of closely related, morphologically highly similar cryptic species. These cryptic species are morphologically indistinguishable, but genetically different [11]. Coexistence of cryptic species in natural environments has been reported at small geographical scales in a broad range of taxa [12,13,14,15], and interactions between cryptic species have been commonly observed [16,17]. Despite this natural coexistence, some laboratory studies have shown that permanent coexistence between closely related species is unlikely under constant environmental conditions [16,18]. Environmental fluctuations may be important in maintaining coexistence, and as a consequence, decreasing amplitudes of daily environmental fluctuations may affect the coexistence of cryptic species.

Cryptic diversity has been frequently observed in coastal nematodes [19]. In the morphospecies *Litoditis marina* [20]; henceforth referred to as *L*. *marina*, formerly known as *Rhabditis marina* or *Pellioditis marina*, at least 10 cryptic species have been found [21]. Species of the *L*. *marina* species complex are typical colonizers of decaying algae and show explosive population growth and rapid colonization/extinction dynamics [22]. These species show concordant molecular divergences at nuclear and mitochondrial loci (COI, ITS, D2D3), but lack single distinctive morphological differences [13,21,23]. Four of them (Pm I, Pm II, Pm III and Pm IV) frequently occur in the littoral zone of the south-western coast and estuaries of The Netherlands [21,24], in which pronounced daily temperature fluctuations are common. Pm I and Pm IV are the most closely related species but cross-breeding between them does not occur [23]. Sympatric occurrence of two or more of these species on decomposing algae is rule rather than exception [21,24]. This coexistence is intriguing since competition between the species exists [16]: Pm I and Pm III proved to be competitively superior to Pm II and Pm IV, but the precise nature of this competition is still unknown, and it may shift from contest to scramble competition depending, among other things, on the abiotic environment. Moreover, facilitation has been demonstrated between these four cryptic species of *Litoditis marina* in experiments using closed microcosms [16]. Dispersal may be one of the mechanisms enabling temporary coexistence [25]. Nevertheless, niche differentiation may also be important, but information about this is still lacking. Differential population responses to salinity [26] and partial differences in their gut bacterial communities (Derycke et al. unpublished) suggest at least some degree of niche differentiation. Moreover, species-specific responses to temperature in a range of 15 to 25°C exist [26]. Salinity can influence these interspecific interactions [16], but the effect of temperature on these interspecific interactions has not been investigated.

In this study, the effect of a temperature regime with daily fluctuations versus a constant temperature on (a) the fitness (here estimated from population size, both for juveniles and adults, [27]) and (b) interspecific interactions of three cryptic species of the *L*. *marina* species complex (Pm I, Pm III and Pm IV) were investigated. Based on previous research [16,21,26], we expected that (a) species which benefit from higher temperatures may have a higher fitness at fluctuating temperature, due to the higher maximal temperature in this regime. Pm III showed a geographical distribution in warmer regions compared with the other species [21] and had a shorter generation time at higher temperature [26], which suggests that Pm III may have a higher fitness at fluctuating temperature. This can also affect species interactions (b), with a dominance of the species with a higher fitness. We can thus expect that Pm III will be dominant over Pm I and Pm IV. However, differences in competitive abilities between the different species can also affect these interactions and temperature fluctuations can have indirect species-specific effects on them. Pm I was found to be competitively superior to Pm IV [16], so we expected Pm IV to have very low abundances or even to go extinct. This study can help us to better understand the coexistence of these closely related species on small spatial scales in natural environments.

## Materials & Methods

### Nematode cultures

Nematodes for the experiments were harvested from monospecific stock cultures in exponential growth phase. Monospecific cultures of three different cryptic species (Pm I, Pm III and Pm IV) were each raised from one single gravid female, obtained from the field (for PmI and Pm III Paulina marsh, Westerschelde, The Netherlands; for Pm IV Lake Grevelingen, The Netherlands) in September 2009, and maintained on sloppy (1%) nutrient:bacto agar media (temperature of 20°C; salinity of 25) with unidentified bacteria from their habitat as food [28]. The temperature of the stock cultures is comparable with the average temperature in the field during summer, while a salinity of 25 approximates the mean salinity in their natural environment.

### Temperature experiments

The experiment comprised three monospecific treatments (respectively M1, M3 and M4 for Pm I, Pm III and Pm IV), one two-species treatment with the two most closely related species: Pm I and Pm IV (D), and one treatment with all three species (T). These treatments (M, D or T) were called the ‘interspecific interaction’ treatment. Three females and two males per cryptic species were incubated in all treatments using an additive design [29]. Hence, total number of nematodes and species varied depending on the treatment (5 nematodes for M, 10 for D and 15 for T). Because intraspecific competition is known to be prominent in these nematodes [25], numbers per species were kept constant in order to be able to elucidate the effect of interspecific competition in all treatments. By adjusting the size of the petri dish, the amount of agar medium and the amount of food (see further), the available space and resources per inoculated nematode in every treatment were kept constant. Monospecific treatments (M1, M3 and M4) were incubated in small petri dishes (inner diameter of 5.4 cm) with 4 mL of 1% bacto agar and 50 μL of a suspension of frozen-and-thawed *Escherichia coli* (strain K12, density of 3x10^10^ cells mL^-1^ [30]). The D treatment was incubated on petri dishes with the same inner diameter of 5.4 cm, but with 8 mL of 1% bacto agar medium and 100 μL of the same *E*. *coli* suspension used for the monospecific treatments. Finally, treatment T contained the three species together on petri dishes with an inner diameter of 8.4 cm, 12 mL of 1% bacto agar and 150 μL of *E*. *coli* suspension. Food was added at the start of the experiment and again after 14 days. Each treatment was replicated nine times (3 replicas at 3 time moments) at two different temperature regimes: a constant air temperature (C) of 20°C and a fluctuating air temperature (F) with 12h of 15°C followed by 12h of 25°C (the change in temperature took approximately half an hour to establish and stabilise). A temperature of 20°C represents average summer temperature, whereas 15°C and 25°C represent fairly common daytime minimum and maximum temperatures during summer [31]. The average temperature was equal in both treatments. All plates were sealed with Parafilm, which prevents evaporation of the agar but still allows oxygen diffusion into the plates. Salinity of the agar medium was 25. The pH of the agar medium was buffered at 7.5–8 with TRIS-HCl in a final concentration of 5mM, which increases the initial salinity by ca. 1.2 units. Cholesterol (100 μL L^-1^) was added as a source of sterols, because nematodes on a purely bacterial diet appear incapable of *de novo* synthesis of specific sterols [32]. After 7, 14 and 21 days, three replicates of every treatment (temperature regime x ‘interspecific interaction’ treatment) were frozen (- 20°C) for later counts and analysis of the assemblage structure and abundance. For the counts of adults and juveniles we used a stereomicroscope for all treatments. Relative quantification of each species in the D and T treatments was based on DNA extraction and qPCR analysis [33] following the same method as in a previous paper [25]. Absolute numbers per species were calculated by multiplying the relative abundances with the total numbers of the plate.

### Statistical analyses

#### a) Effect of temperature on the fitness of the species

Within each species, 3-way ANOVAs were conducted on the numbers of adults and juveniles separately to test the effect of temperature (C or F), interspecific interactions (in case of Pm I and Pm IV: M1/M4, D and T and in case of Pm III: M3 and T) and time. No overall ANOVA with species as factor could be conducted as the data of the different species are not independent from each other within the D and T treatment. ANOVAs were conducted in the statistical software package R [34]. A Tukey’s honestly significant differences test was performed on the significant factors. To achieve normality of the data, a log transformation was performed for data of adults and juveniles of Pm I and for juveniles of Pm IV. For Pm III adults, a PERMANOVA [35] (on the basis of Euclidean distance with 999 permutations) was conducted because the assumptions for normality were not met, even after transformation. A pairwise PERMANOVA was conducted on the significant factors.

#### b) Effect of fluctuating temperature on the interactions between the species

PERMANOVA was also used to investigate the effect of the different temperature regimes, interspecific interactions and time on juvenile and adult assemblage dynamics. This was done by comparing adult and juvenile assemblage compositions in fictitious and real assemblages. Fictitious assemblages were made by summing the abundances of the monospecific treatments (respectively M1 + M4 (= FiD) and M1 + M3 + M4 (= FiT)). These fictitious assemblages are assemblages without interspecific interactions and were compared with the assemblage compositions in which more than one species was present and interspecific interactions were possible (FiD vs. D and FiT vs. T). The relative contribution of each species was the dependent variable, and the independent fixed factors were time (day 7, 14 and 21), temperature regime (C or F) and interspecific interactions (for Pm I and Pm IV: FiD (no interspecific interactions) compared with D (with interspecific interactions), for Pm I, Pm III and Pm IV: FiT (no interspecific interactions) compared with T (with interspecific interactions). Significant terms and interactions were investigated using posterior pair wise comparisons within PERMANOVA. PERMDISP was performed to test the homogeneity of multivariate dispersions (centroid around the mean). A log transformation on the adults was used for the treatment with two species and a fourth root transformation on the juveniles was performed in the treatment with three species to achieve this homogeneity. A SIMPER analysis was used to identify which species primarily accounted for the observed differences. In addition, an ANOVA was conducted to compare the total number of nematodes (regardless of species identity) in FiD with D and FiT with T. A Tukey’s honestly significant differences test was performed on the significant factors.

## Results

### Effect of temperature regime and interspecific interactions on the fitness of the species

**Fig 1:**
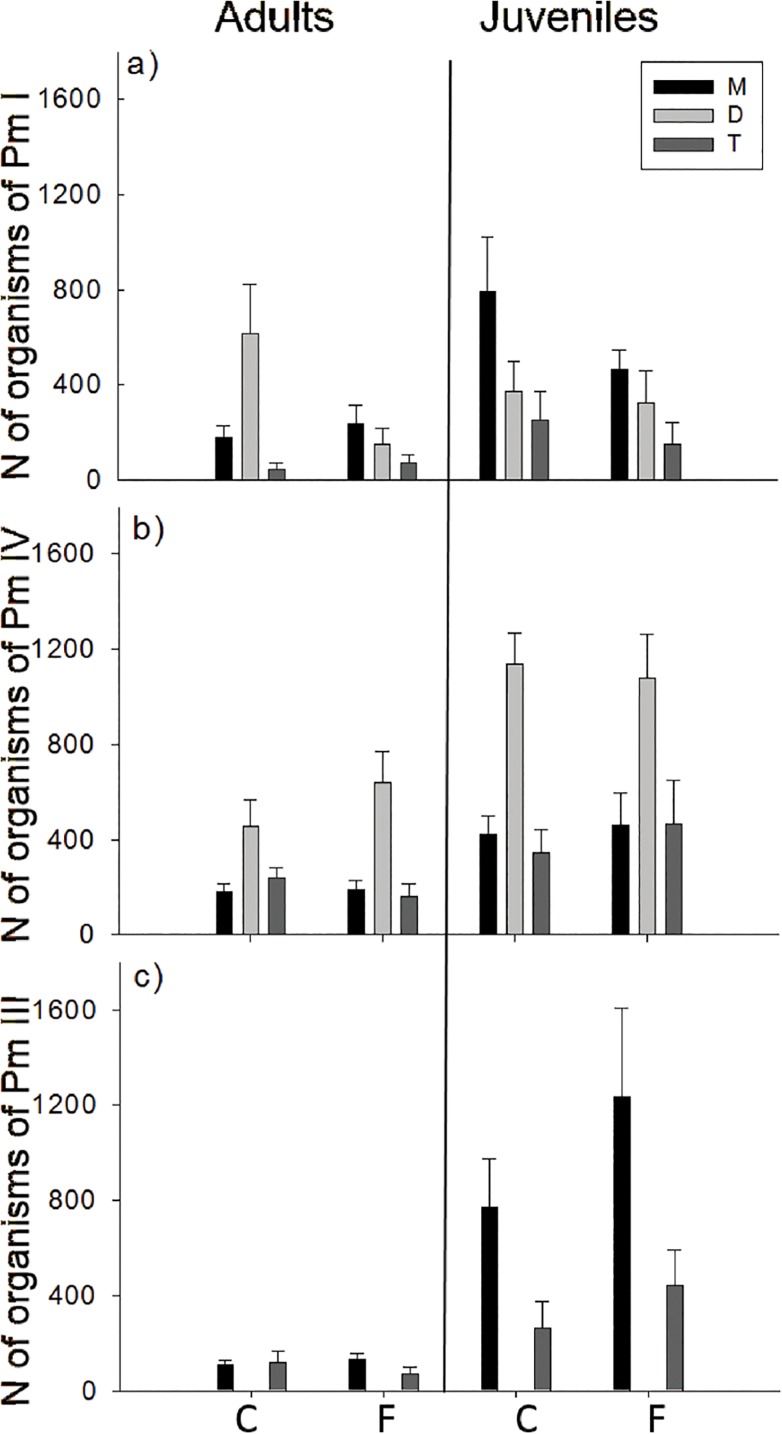
Time-averaged number of nematodes (± SE) of *Litoditis marina* species (adults and juveniles) in the different interspecific interaction treatments (three species: T, two species: D and one species: M) and different temperature treatments (F and C) for (a) Pm I, (b) Pm IV and (c) Pm III.

Temperature regime had no effect on the juvenile or adult abundances of Pm I (Fig 1A). However, the abundance of Pm I adults was influenced by interspecific interactions and time (Table 1). Lower abundances of adults and juveniles were found when all three species were present (treatment T) compared with the two other treatments (all p< 0.03). No significant interaction terms were found (Table 1).

**Table 1:** Statistical results of the effect of temperature regime on fitness.

	Pm I				Pm IV				Pm III			
	Adults		Juveniles		Adults		Juveniles		Adults		Juveniles	
	F	p	F	p	F	P	F	p	F	p	F	p
Temp.	0.13	0.72	1.15	0.29	0.42	0.53	<0.001	0.99	0.25	0.61	6.56	**0.017**
Intersp.int.	19.71	**<0.001**	9.31	**<0.001**	15.93	**<0.001**	16.38	**<0.001**	1.22	0.23	27.61	**<0.001**
Time	6.10	**0.005**	2.95	0.07	5.42	**0.009**	5.33	**0.009**	3.11	0.07	14.90	**<0.001**
Temp.:Intersp.int	1.35	0.27	0.30	0.74	1.69	0.20	0.18	0.83	2.11	0.16	1.33	0.26
Temp:Time	2.96	0.06	0.54	0.59	1.77	0.18	0.34	0.72	2.10	0.15	7.40	**0.003**
Intersp.inter:Time	1.23	0.32	0.79	0.54	1.45	0.24	7.10	**<0.001**	4.40	**0.02**	16.90	**<0.001**
Temp:Intersp.inter: Time	2.05	0.11	0.65	0.63	0.41	0.80	0.13	0.97	2.98	0.07	2.72	0.09

Results of the within-species statistical analyses on fitness (independent factors: temperature (fluctuating vs. constant), interspecific interactions (M, D and T for Pm I and Pm IV; M and D for Pm III) and time; dependent factors: number of adults and juveniles in three cryptic species of *Litoditis marina*). Level of confidence = 95%. Interspec.int. = interspecific interactions; temp. = temperature; p = statistical p value; F = F statistic.

Temperature regime also had no effect on juvenile and adult abundances of Pm IV, while interspecific interactions and time did affect adult and juvenile abundances (Table 1). For this species, however, the highest abundances of adults were found in the D treatment (Pm I and Pm IV together) (p< 0.0001) (Fig 1B). Significant interaction terms were only found for the juvenile abundances between interspecific interaction treatment and time (Table 1). Pm IV juveniles had higher abundances in the D treatment (1121 ± 107.2 juveniles) compared with the M4 treatment (193 ± 69.6 juveniles) only after 7 days. In the T treatment, lower juvenile abundances (92 ± 55.4 juveniles) were present after 21 days compared with the M4 and D treatment (respectively 447 ± 83.1 and 1109 ± 191.7).

Temperature regime did not affect adult abundances of Pm III, but it did affect juvenile abundances (Fig 1C). Numbers of Pm III adults were influenced by the interaction of time and interspecific interaction treatment (Table 1), with more adults after 14 days when Pm III occurred alone (M3: 168 ± 17.7) compared to the treatment where Pm III was incubated together with the two other species (T: 47 ± 23.6) (pairwise PERMANOVA: p = 0.005). This difference was not present after 7 or after 21 days. Time, temperature regime, interspecific interactions treatment, the interaction between time and temperature and the interaction between time and interspecific interactions treatment all had significant influences on the juvenile abundances of Pm III (Table 1). After 14 and 21 days, lower juvenile Pm III abundances were found in the T treatment (respectively 368 ± 160.9 and 287 ± 158.0) compared with the M3 treatment (respectively 1787 ± 354.9 and 1149 ± 176.9). After 14 days, more juveniles were found at the fluctuating temperature (1570 ± 457.5) compared with a constant temperature regime (584 ± 215.9). In Tables 2 and 3, respectively, the average number of nematodes in the different treatments and an overview of the effect of the interspecific interactions on the fitness of all three species can be found.

**Table 2:** Overview of number of nematodes for the different treatments.

	Adults	M		D		T	
**Pm I**		Co	Fl	Co	Fl	Co	Fl
	7 days	48 ± 14	89± 23	37± 18	74± 67	5± 4	55± 29
	14 days	187 ± 94	300± 40	803± 347	78± 51	79± 40	1± 1
	21 days	311± 25	293± 43	1003± 392	353± 142	83± 82	157± 86
	Adults	M		D		T	
**Pm IV**		Co	Fl	Co	Fl	Co	Fl
	7 days	89 ±44	62 ±32	428 ±32	350 ±54	317 ±60	142 ±97
	14 days	257 ±37	300 ±40	590 ±254	994 ±197	228 ±90	316 ±38
	21 days	201 ±11	205 ±48	344 ±263	570 ±259	168 ±85	26 ±15
	Adults	M				T	
**Pm III**		Co	Fl			Co	Fl
	7 days	78±37	90±48			11±11	108±60
	14 days	156±8	179±37			79±39	14±11
	21 days	100±3	136±14			269±84	91±71
	Juveniles	M		D		T	
**Pm I**		Co	Fl	Co	Fl	Co	Fl
	7 days	99±45	286±46	118±21	67±21	92±54	52±18
	14 days	986±463	818±288	537±330	606±280	150±93	292±287
	21 days	1304±53	634±219	463±200	526±335	380±341	107±80
	Juveniles	M		D		T	
**Pm IV**		Co	Fl	Co	Fl	Co	Fl
	7 days	169±64	217±140	1030±124	1212±348	442±108	462±159
	14 days	555±75	818±288	1220±403	978±334	454±246	886±451
	21 days	537±149	356±63	1162±160	1056±394	140±114	44±10
	Juveniles	M				T	
**Pm III**		Co	Fl			Co	Fl
	7 days	88±16	74±15			287±148	525±383
	14 days	1018±189	2554±71			150±93	586±271
	21 days	1213±288	1086±264			360±323	214±122

Number of nematodes ± SE (adults or juveniles) over time for the different temperature treatments (Co = constant; Fl = fluctuating) and interspecific interaction treatments.

**Table 3:** Overview of the effect of the interspecific interactions on the fitness.

	Adults		Juveniles	
	D	T	D	T
Pm I	0*	0	0	-
Pm IV	+	0	+	0 ^b^
Pm III	NA	0^a^	NA	- ^c^

Effect of interspecific interactions on population abundance (adults and juveniles) of the different cryptic species of *Litoditis marina* (Pm I, Pm III and Pm IV) in the D and T treatment compared with the M treatment (0 = statistically no differences;-: lower abundance compared with M; +: higher abundance compared with M).

*: At constant temperature a positive effect for Pm I adults occurred

a: at 14 days a negative effect occurred

b: at 21 days a negative effect occurred

c: at day 7 no difference was found

### Assemblage composition and dynamics

**Fig 2:**
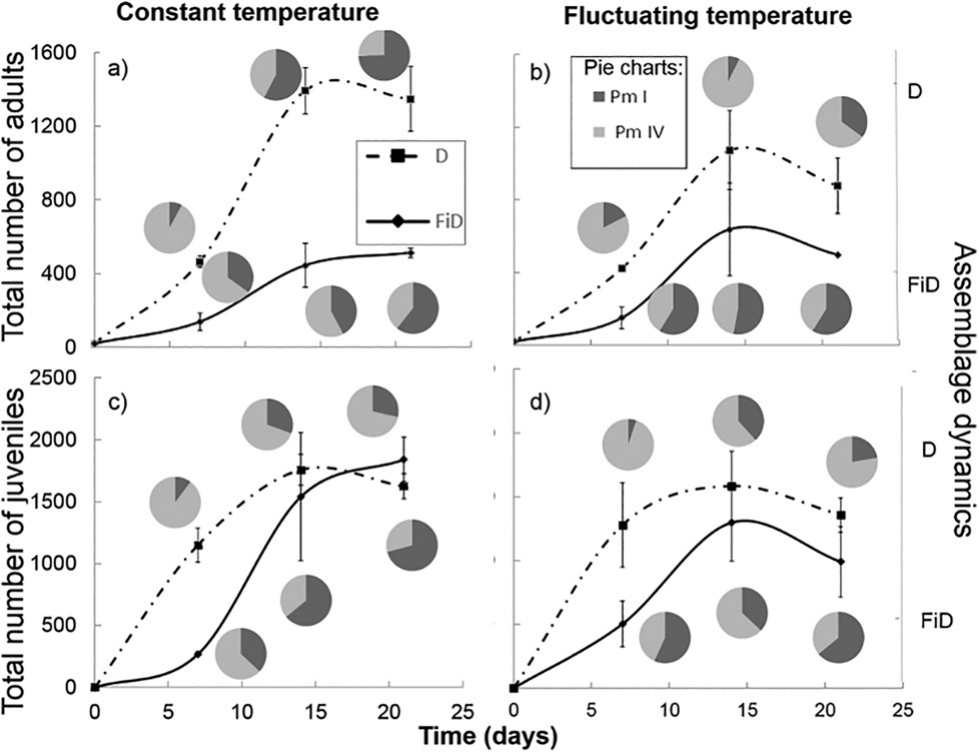
Total number of nematodes (adults: a + b, juveniles: c + d) over time in the different temperature treatments (constant temperature: a + c, fluctuating temperature: b + d) with assemblage dynamics at the different sampling times (three pie charts correspond with following time moments: 7, 14 and 21 days): upper pie charts for the D treatment (Pm I and Pm IV with interspecific interactions), lower pie charts are the dynamics in the FiD treatment (Pm I and Pm IV without interspecific interactions).

In the treatment with two species, total abundances of adults and juveniles (regardless of species) were affected by time and interspecific interactions treatment (respectively F_2,24_ = 24.30, p<0.001 and F_1,24_ = 50.03, p<0.001 for adults and F_2,24_ = 10.55, p<0.001 and F_1,24_ = 7.10, p = 0.014 for juveniles), but not by temperature. Lowest abundances of nematodes occurred after 7 days. They did not differ between 14 and 21 days. Much higher numbers of nematodes were observed in the D treatment compared with the FiD treatment (respectively 930 ± 171.6 vs. 97 ± 83.9 adults and 1459 ± 95.1 vs. 1074 ± 274.6 juveniles (Fig 2)). Comparing adult assemblage dynamics of Pm I and Pm IV between the D and FiD treatments showed significant effects of interspecific interactions, time and the interaction of interspecific interactions with temperature regime on the assemblage composition (Table 4). Pm I became dominant over Pm IV in the D treatment at the end of the experiment at a constant temperature, but the opposite was true at a fluctuating temperature. In the fictitious treatment (FiD), however, Pm IV was not dominant over Pm I at this fluctuating temperature (Fig 2A). For juvenile assemblage dynamics, only time and interspecific interactions were significant (Table 4, Fig 2B). A clear effect of interspecific interactions was shown, with Pm IV juveniles being dominant over Pm I in the D treatment compared with the FiD treatment independent of temperature regime.

**Table 4:** Statistical results of the effect of temperature regime on assemblage dynamics.

	D compared with FiD				T compared with FiT			
	Adults		Juveniles		Adults		Juveniles	
	F	p	F	p	F	p	F	p
Temp.	2.88	0.09	0.99	0.39	0.85	0.50	3.66	**0.019**
Intersp.int.	9.27	**0.001**	14.59	**0.001**	6.44	**0.004**	14.42	**0.001**
Time	5.38	**0.002**	3.88	**0.01**	4.60	**0.004**	9.01	**0.001**
Temp.:Intersp.int.	4.36	**0.03**	0.62	0.51	0.85	0.44	0.83	0.46
Temp.:Time	1.24	0.30	0.70	0.60	1.05	0.39	3.79	**0.002**
Interspec.int.:Time	1.01	0.40	1.26	0.28	3.17	**0.01**	7.69	**0.001**
Temp.:Intersp.int.:	1.22	0.33	0.76	0.49	1.22	0.30	1.34	0.24
Time								

Results of the two 3-way PERMANOVA analyses on interspecific interactions (independent factors: temperature (constant vs. fluctuating), interspecific interactions (fictitious vs. real populations) and time), dependent factors: adult and juvenile assemblage compositions)) for experiments with two species (D vs. FiD) and three species (T vs. FiT). Level of confidence = 95%. Interspec.int. = interspecific interactions; temp. = temperature; p = statistical p value; F = F statistic.

**Fig 3:**
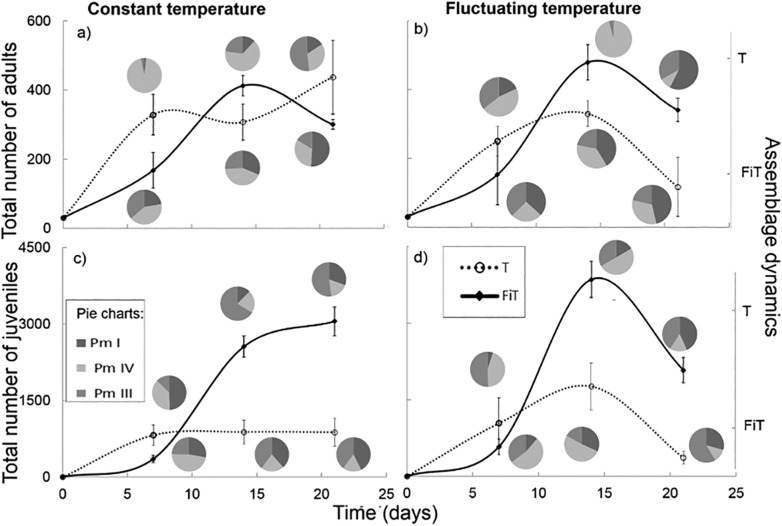
Total number of nematodes (adults: a + b, juveniles: c + d) over time in the different temperature treatments (constant temperature: a + c, fluctuating temperature: b + d) with assemblage dynamics at the different sampling times (three pie charts correspond with following time moments: 7, 14 and 21 days): upper pie charts for the T treatment (Pm I, Pm IV and Pm III with interspecific interactions), lower pie charts are the dynamics in the FiT treatment (Pm I, Pm IV and Pm III without interspecific interactions).

In the treatments with three species, total numbers of adults (regardless of the species) were affected by time, the interaction between time and interspecific interactions treatment as well as the interaction between interspecific interactions treatment and temperature (respectively F_2,24_ = 6.88, p = 0.004, F_2,24_ = 4.73, p = 0.019 and F_1,24_ = 5.05, p = 0.034), with lower numbers of adults in the T treatment compared with the FiT treatment at fluctuating temperature at the end of the experiment (Fig 3). In the T treatment, more adults were found at the constant temperature compared with the fluctuating temperature (respectively 358 ± 43.2 and 232 ± 43.0 adults). Total abundances of juveniles (regardless of species) were affected by time, interspecific interactions treatment, temperature (respectively F_2,24_ = 17.71, p<0.001, F_1,24_ = 17.35, p<0.001 and F_1,24_ = 5.89, p = 0.023), the interaction between time and interspecific interactions treatment, and the interaction between time and temperature (respectively F_2,24_ = 14.95, p<0.001 and F_2,24_ = 8.46, p = 0.002). Abundances of juveniles differed between the T and FiT treatment after 14 days and 21 days, with very low numbers of juveniles in the T treatment. Only at day 14, numbers of juveniles were higher in the fluctuating temperature regime compared with the constant temperature (Fig 3). For the assemblage dynamics, no effect of temperature regime on the adult assemblages was found. There was an effect of time, interspecific interactions (T vs FiT) and the interaction of time and interspecific interactions (Table 4) on the adult assemblages. Differences in assemblage dynamics were found between the FiT and the T treatment at every time moment (Fig 3). SIMPER analysis showed that Pm I was the main responsible for the dissimilarity between these treatments. Pm I was less abundant in the T treatment than expected based on the FiT-treatments, in which Pm I became dominant after 14 days (Fig 3A and 3B), while Pm IV was the most abundant species in the T treatment after 7 and 14 days and Pm III after 21 days. Moreover, juvenile assemblage composition was also influenced by time, interspecific interactions, temperature, the interaction of time and interspecific interactions and the interaction of time and temperature (Table 4, Fig 3C and 3D).

The assemblage dynamics differed between the FiT and T treatment and corresponded well with those observed in the adults. After 7 days, Pm I juveniles were less dominant in the T treatment than expected based on the FiT treatment, and after 14 days Pm IV was the most abundant species in the T treatment. After 21 days Pm III juveniles became more abundant compared with the other time moments (Pm III contributions to differences between time moments: all > 47.63%). The difference between the FiT and T treatment was mainly due to Pm IV (65.23% contribution to the dissimilarity). Temperature regime had an effect on day 14, when Pm III juveniles were more abundant at constant temperature compared with the fluctuating temperature (Pm III contributed 62.11% to this dissimilarity).

## Discussion

The results of this study demonstrate that interspecific interactions rather than temperature regime governed assemblage dynamics of species mixtures. The effect of daily temperature fluctuations in the tested range on the fitness of cryptic species of *Litoditis marina* is limited and species-specific. However, temperature regime did affect certain interactions between species.

### Fluctuating temperature and interspecific interactions affect fitness in a species-specific way

Fluctuating temperature had no differential effect compared with constant temperature on the fitness of Pm I and Pm IV populations. However, the population fitness of Pm III was affected by temperature regime: higher juvenile abundances occurred after 14 days under fluctuating temperature. This could be the result of a positive effect of the maximum temperature on life-history traits, such as reproduction and development time [36]. Experiments at constant temperatures have indeed shown that Pm III performs better at 25°C than at 15°C [26], and that this effect is more pronounced than in the other cryptic species. Moreover, phylogeographic data show the presence of Pm III in regions with higher average temperatures, where the other species were absent [21]. This may indicate that Pm III is better adapted to higher temperatures than the other cryptic species, while it does not perform worse than the other species at lower temperatures (15°C and 20°C [26]). However, the higher abundance of Pm III juveniles at fluctuating temperature was a transient feature only found after 14 days, which could also point to a stress response (different conditions compared with the stock culture). Hence, temperature regime had only limited effects on population fitness of any of the three *Litoditis* species in monoculture.

In contrast, interspecific interactions clearly influenced the fitness of all three species, both positively and negatively. Decreased population sizes are the result of interspecific competition between the species, which can be due to reductions in survival, growth or fecundity [37]. Competition was asymmetrical, mainly affecting the abundances of Pm I and Pm III juveniles but not those of Pm IV. Asymmetrical competition has also been found among other bacterial-feeding free-living nematodes [38,39]. In contrast, Pm IV appeared to benefit from the presence of Pm I (D treatment), suggesting some sort of facilitative interaction [40]. Over time, the interspecific interaction effect sometimes changed, which indicates that population dynamics are still changing and that longer-term studies can be important to properly predict the outcome of those interactions.

### Fluctuating temperature alters some interspecific interactions

Temperature fluctuations altered interactions between Pm I and Pm IV in the two-species treatment but not in the three-species treatment. Pm I and Pm IV are phylogenetically more closely related to each other than to other cryptic *Litoditis marina* species, and we therefore expected stronger competition between them according to the competition-relatedness relationship [10]. Indeed, in a previous competition experiment with four cryptic species in closed microcosms under constant environmental conditions, Pm IV was completely outcompeted [16]. The current experiment contradicts our prediction: Pm I and Pm IV were able to coexist in high abundances, even under constant temperatures, suggesting that Pm I was not the main competitor of Pm IV in our earlier experiment, and/or that the presence of additional species changes the type of their interaction. In fact, at a constant temperature, both Pm I and Pm IV attained higher population abundances when they occurred together (without the third species, D treatment) (for adults and juveniles in Pm IV, only for adults in Pm I), suggesting a sort of facilitative mutualism [40,41]. Higher total nematode densities at the start of the experiment can potentially affect bacterial growth and abundance through grazing or mucus production [42] and could thus have increased food availability and enhanced nematode growth in both species. At fluctuating temperature, the facilitative effect was still pronounced for Pm IV, but disappeared for Pm I. Pm IV now became more abundant than Pm I, pointing at a facilitative commensalism, with a positive effect of Pm I on Pm IV, and no effect of the presence of Pm IV on Pm I. Temperature regime thus altered the interaction between these two species from a sort of mutualism to commensalism, demonstrating that interactions between the species can change depending on the abiotic environment [43]. Such environmental impacts on species interactions could result from species-specific responses to the abiotic environment. However, in this experiment, no significant differences in fitness were found in the monospecific treatments at fluctuating temperature compared with the constant temperature. Experiments on their life history at constant temperatures (15°C, i.e. the lowest temperature in our F treatment, 20°C and 25°C, i.e. the highest temperature in our F treatment) revealed no obvious differences in generation time, reproduction rate or total population development between these two *L*. *marina* species (Pm I and Pm IV) [26], suggesting that differences in their life histories at these temperatures are negligible. Nevertheless, some studies on fish and butterflies have shown that fluctuating compared to constant temperatures caused shorter development times [44], and we did not include development or generation time as life-history traits in our present experiment. Hence, further investigation on the effect of fluctuating temperature on generation time is needed to check if the difference in interactions is the result of differences in life history. Another possibility is a direct effect on the interspecific behaviour of the two species with the abiotic factors having an effect on the way species interact with each other [43], for instance by influencing interference behaviour [45]. Additionally, the result of the interspecific interactions was not fully consistent among adults and juveniles: whereas adult abundances of both Pm I and Pm IV were higher in the combination treatment (D, a sort of mutualism), only Pm IV juveniles were more abundant in the D than in the M treatment (facilitative commensalism). Processes as maturation, reproduction and mortality could be differentially influenced at each stage of the individual by interspecific competitive interactions [46]. Valiente-Banuet and Verdú [41] demonstrated in plants that interactions can alter along their development and/or in response to temporal fluctuations of the environment. In this experiment, an effect of both could be found: differential interactions between adults and juveniles of the different species were found under certain abiotic conditions. Juveniles have often been demonstrated to be more sensitive to various kinds of environmental stress than adults [47], which may contribute to such differential interactions.

The interactions between the species changed when three species were present (T treatment), and temperature did not alter these interactions; however, the total number of nematodes (regardless of species) was affected by the temperature regime. Over time, the dominance of the species changed in adult and juvenile assemblages in both temperature regimes: in the beginning, Pm IV was the most abundant species, whereas after 21 days Pm III became the most abundant one. This suggests that the community was still changing after three weeks. For Pm III juveniles this dominance occurred faster for the constant temperature (already at 14 days) compared with the fluctuating temperature. However, Pm I and Pm III showed lower abundances in the T treatment compared to the respective monospecific treatments (M1 and M3) (Fig 1), which can be the result of competition between these two species. No effect of Pm I and Pm III on Pm IV was found. These results are in conflict with a previous study [16] in which the same species, together with a fourth one (Pm II), were simultaneously inoculated into closed microcosms. In that experiment, Pm II and Pm IV adults went extinct after 35 days at a constant temperature of 20°C, whereas no substantial effect of competition on Pm I and Pm III was evident. Perhaps the time frame of the present experiment was too short for this competitive effect to become manifest. The fact that Pm IV juvenile abundance dropped after 21 days in the T treatment compared to the M4 treatment may point to this explanation. Alternatively, the extinction of Pm IV in that previous experiment could have mainly resulted from competition with Pm II and/or Pm II could have changed the interactions between the other species. It seems that there are complex interactions between the species, which are not just the sum of their separate pairwise interactions. As a result, a competitively intransitive network, in which species’ abilities cannot be ranked in a hierarchy [48], exists in this cryptic species complex. An addition of one species to a community can change all existing interactions between the others. One species can alter the effect that another species has on a third one, and thus pairwise species interactions are influenced by the presence and density of other species in the community. These indirect effects may importantly affect the success of a species [49]. Moreover, total number of juveniles was lower when interspecific interactions occurred, again pointing to the fact that juveniles may be more sensitive to stress [47]. Altough there was no clear effect of temperature regime on the assemblage dynamics, fluctuating temperature had an effect on the total abundances of nematodes over time (regardless of species) in the T treatment, with a decrease in abundances by the end of the experiment compared with the constant temperature. This could point out that the competitive interactions will be more severe for all species at fluctuating temperature, without affecting the relative contribution of each species. Salinity already proved to have an effect on the strength of the interactions between *Litoditis* species [16], showing that differences in abiotic parameters can change the strength of interspecific interactions.

## Conclusions

The results of this experiment show that a competitively intransitive network between the cryptic species of *Litoditis marina* exists and daily temperature fluctuations can alter these interactions. Fluctuating temperature only had a small effect on the fitness of one of the three cryptic species studied here, but interspecific interactions can change or get weaker depending on the temperature regime. The results indicate that there is a complex interaction between abiotic and biotic factors, and that temperature fluctuations may change interspecific interactions, depending on the assemblage dynamics. The outcome of interactions cannot be easily predicted, but in natural situations these different regulators—biotic (interspecific interactions) and abiotic (temperature)- can alter the outcome of the interactions between species and temperature fluctuations may facilitate coexistence.
